# Discovery of the first dual inhibitor of the 5-lipoxygenase-activating protein and soluble epoxide hydrolase using pharmacophore-based virtual screening

**DOI:** 10.1038/srep42751

**Published:** 2017-02-20

**Authors:** Veronika Temml, Ulrike Garscha, Erik Romp, Gregor Schubert, Jana Gerstmeier, Zsofia Kutil, Barbara Matuszczak, Birgit Waltenberger, Hermann Stuppner, Oliver Werz, Daniela Schuster

**Affiliations:** 1 Institute of Pharmacy/Pharmaceutical Chemistry and Center for Molecular Biosciences Innsbruck (CMBI), University of Innsbruck, Innrain 80-82, A-6020 Innsbruck, Austria; 2 Institute of Pharmacy/Pharmacognosy and Center for Molecular Biosciences Innsbruck (CMBI), University of Innsbruck, Innrain 80-82, A-6020 Innsbruck, Austria; 3 Chair of Pharmaceutical/Medicinal Chemistry, University of Jena, Philosophenweg 14, 07743 Jena, Germany; 4 Laboratory of Plant Biotechnologies, Institute of Experimental Botany AS CR, Rozvojova 263, Prague 6 - Lysolaje, Czech Republic

## Abstract

Leukotrienes (LTs) are pro-inflammatory lipid mediators derived from arachidonic acid (AA) with roles in inflammatory and allergic diseases. The biosynthesis of LTs is initiated by transfer of AA via the 5-lipoxygenase-activating protein (FLAP) to 5-lipoxygenase (5-LO). FLAP inhibition abolishes LT formation exerting anti-inflammatory effects. The soluble epoxide hydrolase (sEH) converts AA-derived anti-inflammatory epoxyeicosatrienoic acids (EETs) to dihydroxyeicosatetraenoic acids (di-HETEs). Its inhibition consequently also counteracts inflammation. Targeting both LT biosynthesis and the conversion of EETs with a dual inhibitor of FLAP and sEH may represent a novel, powerful anti-inflammatory strategy. We present a pharmacophore-based virtual screening campaign that led to 20 hit compounds of which 4 targeted FLAP and 4 were sEH inhibitors. Among them, the first dual inhibitor for sEH and FLAP was identified, *N*-[4-(benzothiazol-2-ylmethoxy)-2-methylphenyl]-*N*’-(3,4-dichlorophenyl)urea with IC_50_ values of 200 nM in a cell-based FLAP test system and 20 nM for sEH activity in a cell-free assay.

In recent years, the “one-drug-hits-one-target” approach has essentially lost ground. Several successfully marketed drugs were shown to actually affect a multiplicity of targets in retrospective. A prominent example is acetylsalicylic acid, which was initially believed to interact solely with cyclooxygenases (COXs), but actually also interferes, among others, with mitogen-activated protein kinases and nuclear factor κB^1^. Several natural products with so-called privileged structures often affect a certain disease not only via a single target but rather interfere with pathologies at a variety of points of attack, with particular relevance for inflammation^2^. Drugs with polypharmacological modes of action were shown to be advantageous over combination therapy as they exert lower incidences of side effects and often lead to more resilient therapies^3^. Therefore, the rational development of chemical structures that contain fragments to inhibit multiple targets, so-called designed multiple ligands (DML), has emerged as a highly interesting field of research with promise for better pharmacotherapies^3^.

Computational approaches offer a valuable means for rational, tightly structured analysis of target families^4^ and can be used for drug design focusing on multiple targets. Pharmacophore modeling allows to condense the functionalities of active compounds towards target-specific interaction patterns^5^. By combining multiple pharmacophore models for different targets in a virtual screening, it is indeed possible to discover structures that contain fragments to affect two or more targets^6^.

A key biochemical pathway for targeting multiple inflammatory conditions is the arachidonic acid (AA) cascade. AA is released from membrane phospholipids by cytosolic phospholipase A2 (cPLA2) and further transformed via at least three separate routes: COX, lipoxygenases (LOs), and cytochrome P450 (CYP450) pathways. Prostaglandins (PGs) and thromboxane are formed via the COX pathway, whereas pro-inflammatory leukotrienes (LTs) but also specialized pro-resolving lipid mediators (SPMs, i.e. lipoxins, resolvins, protectins, and maresins) are generated via the 5-LO and related LO cascades. Finally, CYP450 monooxygenases transform AA to anti-inflammatory epoxyeicosatrienoic acids (EETs), which are further converted to dihydroxyeicosatetraenoic acids (di-HETEs) by soluble epoxide hydrolase (sEH)^7^,^8^. Due to the multitude of pro-inflammatory and pro-resolving mediators produced from one substrate (i.e. AA) in at least these three branches, blocking a single branch by a selective drug may cause redirection/shunting and amplification of alternative pathways, eventually even connected to increased adverse effects. Therefore, smart polypharmacological approaches promise better effectiveness with even fewer side effects^7^.

5-LO is a reasonable target to inhibit the biosynthesis of pro-inflammatory LTs. LTB4 and cysteinyl-LTs are derived from LTA4, a mediator that is synthesized from AA by 5-LO. While several 5-LO inhibitors were developed, only zileuton has become a marketed drug so far^9^. *In cellulo*, 5-LO requires the 5-LO-activating protein (FLAP) for formation of LTA4^10^. FLAP, a nuclear membrane-anchored protein with apparently no enzymatic activity, is supposed to transfer liberated AA to 5-LO. Pharmacological or genetic inhibition of FLAP abolished 5-LO product formation *in vivo*^11^,^12^.

Only comparatively few chemical scaffolds have been reported as FLAP inhibitors such as MK-886, an indole-class compound^13^, and a series of quinolone-based inhibitors^14^, but in both cases research was discontinued. Recently however, FLAP has regained attention as a drug target, most prominently with GSK2190915, a novel promising indole-based derivative that completed phase II trials for the treatment of asthma^15^. In 2015 research on FLAP inhibitors received another boost with the development of a series of oxadiaozole-containing FLAP inhibitors, shown by Takahashi *et al*.^16^ and the discovery of AZD6642, another potent FLAP inhibitor^17^.

Due to the notion that the arterial wall of hypercholesterolemic patients is in a state of chronic inflammation, LTs have also been implicated in cardiovascular conditions, and the FLAP coding gene ALPOX5AP was revealed as a key gene for coronary heart disease in familial hypercholesterolemia patients^18^,^19^.

Upon formation of EETs from AA by CYP ω-oxidases, they are rapidly degraded by sEH to the inactive corresponding di-HETEs^20^. Therefore, sEH inhibition may lead to elevated EET levels thereby counteracting inflammation. In contrast to FLAP inhibitors, a broad variety of sEH inhibitors is found in the literature. They all display highly specific sEH interaction patterns around an amide or a urea functionality and are therefore ideally suited for pharmacophore modeling. In a recent publication, we presented a series of sEH pharmacophore models with the ability to prospectively identify new sEH inhibitors^21^.

Targeting both LT synthesis via inhibition of 5-LO and the conversion of EETs by suppressing sEH with a combination of two inhibitors led to an enhanced anti-inflammatory effect compared to single treatment^22^. Recently, a series of dual sEH/5-LO inhibitors, discovered by a DML approach, were reported with promising results^23^. FLAP was shown to assist 5-LO at the nuclear membrane also in the formation of anti-inflammatory lipoxin A4 and resolvin D1^24^, while cytosolic 5-LO (distant from FLAP and the nuclear membrane) was suggested to form lipoxin A4 in a FLAP-independent manner^25^. Based on promising results from pre-clinical and clinical studies with FLAP inhibitors versus 5-LO inhibitors, FLAP might be the superior target to interfere with LT biosynthesis^26^. But so far, there are no dual sEH/FLAP inhibitors available. In this study, we pursued a pharmacophore model-based virtual screening approach leading to potentially novel, powerful compounds that target sEH and FLAP with anti-inflammatory potential.

## Results

We first focused on the development and validation of ligand-based pharmacophore models for FLAP based on published FLAP inhibitors. Our aim was to combine the new FLAP inhibitor models with the previously developed sEH inhibitor models^21^ to identify potential dual FLAP/sEH inhibitors in a prospective virtual screening.

The FLAP models were generated based on a concise dataset of 11 active compounds from literature. Since FLAP “activity” can only be determined via analysis of cellular 5-LO product formation and is therefore difficult to distinguish from 5-LO activity itself, it was crucial to comprise the dataset only from compounds that were experimentally verified as specific FLAP inhibitors, either by use of crystallization, radio ligand assays, or by unambiguously excluding 5-LO as a target. An overview of the dataset is given in the Supplementary Information Table S1, compounds S1–S11.

**Figure 1:**
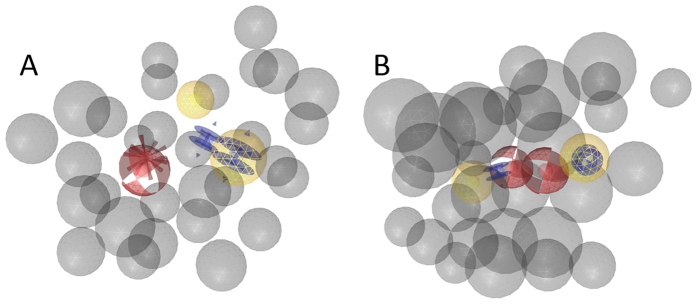
(**A**) Pharmacophore model FLAP1: This model was generated by aligning compounds S10 and S11 (Table S1, Supplementary Information). It consists of two aromatic features (blue rings), two hydrophobic features (yellow spheres), a hydrogen-bond acceptor feature (HBA, red sphere), a negative ionizable feature (red star), and a coat of exclusion volumes (X-vols, grey spheres). **(B)** Model FLAP2. It consists of two hydrophobic features, two aromatic features, two HBA features, and an X-vols coat.

Two ligand-based pharmacophore models were generated as follows:

Model **FLAP1** (see Fig. 1A) was generated by aligning compounds S**10** and S**11**, two indole-based FLAP inhibitors that are distinguished by a quinoline moiety. The model was refined and finally found 8 out of the 11 FLAP inhibitors (including the molecules it was generated from) as described in the Supplementary Information.

The selectivity of the models was investigated by screening them against a drug-like virtual library (12,775 compounds)^27^, which yielded 138 hits for this model.

The second model **FLAP2** (Fig. 1B) was based on an alignment of supplementary compounds S7–S9, two substituted 2,2-bisaryl-bicycloheptanes (S7, S9) and one 1,1-bisaryl-cyclopentane (S8)^28^. The model was refined and finally found 9 out of the 11 FLAP inhibitors (including the structures it was generated from) and 3 hits in the virtual library.

Together, the two models found all 11 active compounds within the dataset. For experimental validation, both models were set to screen the commercial SPECS virtual library (www.specs.net). FLAP1 retrieved 204 virtual hits, while FLAP2 found 833. To ensure structural diversity among the hits, they were clustered into ten different structural categories and from each cluster the compound with the highest geometric pharmacophore fit value (assigned by LigandScout) was selected for testing. This lead to a total of 20 compounds selected for experimental testing.

**Figure 2:**
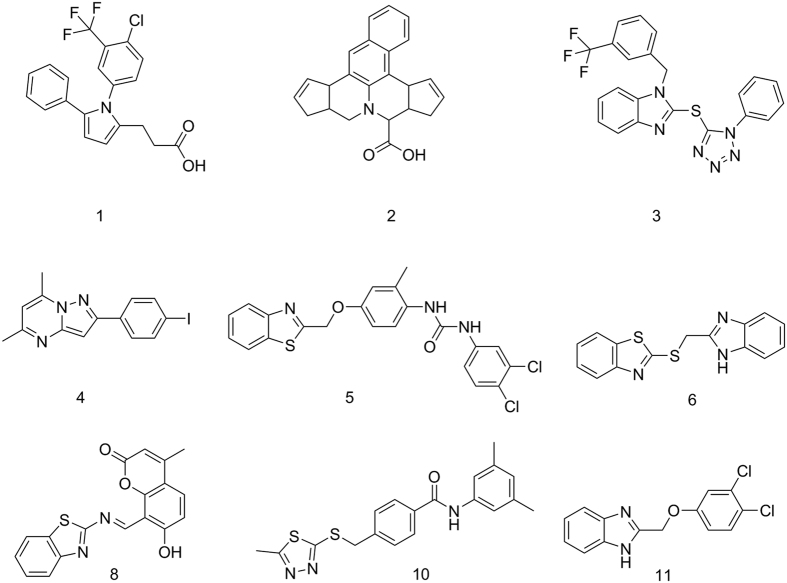
Chemical structures of bioactive compounds.

**Figure 3:**
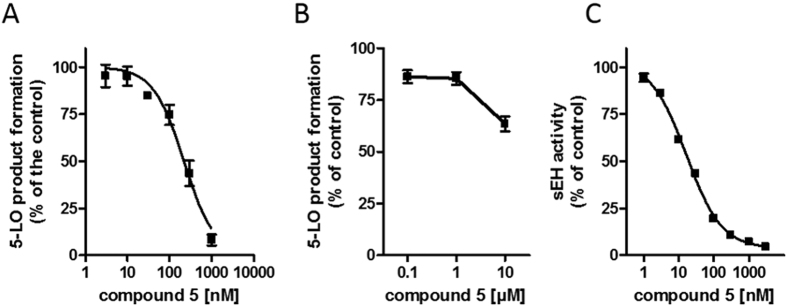
Concentration-response curves for inhibition of FLAP-dependent 5-LO product formation and sEH activity. (**A**) FLAP-dependent 5-LO product formation in intact PMNL (**B**) inhibition of 5-LO activity, cell-free assays, and (**C**) inhibition of sEH activity by compound **5**. Data, means ± SEM, n = 4.

The bioactivity of the selected compounds against FLAP was evaluated using a well-established bioassay, based on intact human neutrophils that were pre-incubated with the test compounds (10 min) and stimulated with Ca^2+^-ionophore A23187 for another 10 min, followed by RP-HPLC analysis of formed 5-LO products^29^. To exclude interference of the hits with 5-LO and thus, to discriminate between FLAP and direct 5-LO inhibition, the compounds were tested for suppression of isolated 5-LO in a cell-free assay (in the absence of FLAP). Out of the 10 identified hits by model FLAP1, three compounds (Fig. 2) were active on FLAP: **1**, a substituted pyrrole, **2**, a dicyclopentanaphthoquinolizine (47.8 (**1**) and 59.9% (**2**) remaining 5-LO product formation at 10 μM, respectively), and **3**, a substituted benzimidazole (23.5% remaining 5-LO product formation at 10 μM). However, **2** also inhibited 5-LO directly in the cell-free assay and thus may not necessarily act on FLAP. Together, these data reflect a true positive hit rate of 20% for model FLAP1. For model FLAP2, three out of 10 compounds (**4**, **5**) were significantly active against FLAP (i.e., <60% remaining 5-LO product formation at 10 μM, without affecting 5-LO directly) leading to a hit rate of 30%. Strikingly, compound **5** turned out as a highly potent inhibitor of 5-LO product biosynthesis (IC_50_ = 200 nM, Fig. 3A) in intact cells that is mediated by FLAP. Note that **5** failed to inhibit isolated 5-LO in the cell-free assay (Fig. 3B), supporting **5** as FLAP inhibitor. Compounds 5 and 6 were hardly active against FLAP.

Seven compounds that were identified by model FLAP2 were predicted as potential sEH inhibitors. A complete list of the selected structures and the respective fitting models can be found in Table S2 in the Supplementary Information. A cell-free assay was applied to determine the inhibitory potency of the compounds against human sEH^21^. Three of the compounds (**7**, **8** and **9**) did not inhibit sEH (IC_50_ > 30 μM, Table 1) and 3 compounds (**10**, **11** and **6**) moderately inhibited sEH activity with IC_50_ of 3 to 12 μM (Table 1). Only **5** potently interfered with sEH (IC_50_ = 20 nM, Fig. 3C) being even somewhat superior over AUDA (IC_50_ = 30 nM), a reference sEH inhibitor^30^. Together, our two ligand-based pharmacophore model virtual screening campaign identified **5** as potential dual FLAP/sEH inhibitor and biological evaluation revealed highly potent, dual inhibition of FLAP and sEH with IC_50_ values of 200 and 20 nM, respectively.

## Discussion

Exploiting pharmacophore-based models and virtual screening, we identified the first dual inhibitor for sEH and FLAP with high potency in the nanomolar range. Both model FLAP1 and model FLAP2 were able to identify two active compounds out of ten tested virtual hits that revealed significant bioactivities. A detailed description of the hits and their orientation in the pharmacophore models is given in the Supplementary Information. Of note, the two virtual hit compounds identified by model FLAP2 also inhibited sEH (**5** and **6**), and out of the 10 test compounds, two more were active on sEH (**10** and **11)** without activity against FLAP.

**Figure 4:**
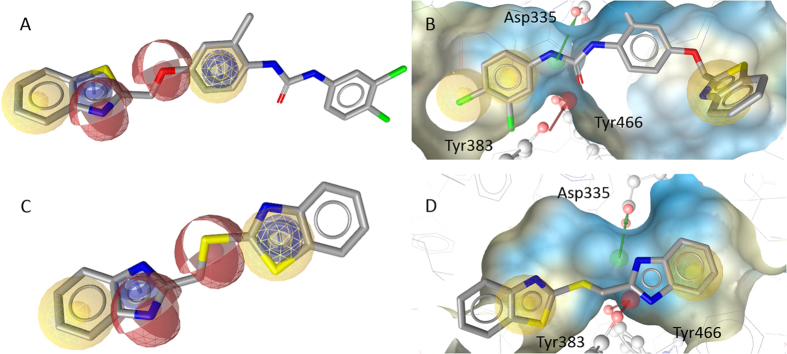
Compound **5** mapped on model FLAP2 (**A**) and within the binding pocket of sEH mapping pharmacophore model 1 based on pdb entry 3ant^43^ (**B**), compound **6** mapped on model FLAP2 (**C**) and with the sEH model 4 based on pdb entry 3i1y^44^ (**D**).

The most intriguing structure discovered in this work is the dual FLAP/sEH inhibitor **5** that potently interfered with both sEH (IC_50_ = 20 nM, Fig. 3) and FLAP (IC_50_ = 200 nM, Fig. 3). To immediately identify such a potent compound by a virtual screening campaign is remarkable and unusual, highlighting the fortunate success of our efforts. Compound **5** contains an urea moiety that is supposedly responsible for the high activity against sEH (see Fig. 4B) and enables the characteristic HBA and HBD interactions with ASP335, Tyr383, and Tyr466^19^. Urea containing structures have been shown to be highly effective on sEH^21^. The requirements for FLAP inhibition are fulfilled by the benzothiazole moiety of the molecule, covering one hydrophobic, one aromatic, and a HBA feature of the pharmacophore. The second HBA feature is mapped on the ether function connected to the phenyl ring, which covers the second hydrophobic and aromatic feature (Fig. 4A). The urea group and the chlorine substituted phenyl group are not essential for binding in the FLAP model, but do not hinder either, thus enabling the molecule to be dually active against FLAP and sEH at such low concentrations.

Compound **6** was only hardly active against FLAP and sEH even at high concentrations (IC_50_ = 18 and 11.5 μM, respectively). It is composed of a benzothiazole and a benzimidazole connected by a thioether. Similar to the benzothiazole of **5**, the benzimidazole of **6** covers one hydrophobic, one aromatic, and one HBA feature of model FLAP2. The second HBA is mapped on the thioether and the second heterocycle also covers one aromatic and hydrophobic feature (see Fig. 4C). In the binding pattern for sEH, the crucial HBA and hydrogen bond donor (HBD) features are covered by the benzimidazole, while the two hydrophobic features are mapped each on one of the heterocycles (Fig. 4D). Although the activity of **6** is not as remarkable as that of **5**, and benzimidazoles are known to inhibit sEH^31^,^32^,^33^, it represents an interesting new scaffold for FLAP, due to its low molecular weight and comparatively simple structure. The ligand efficiency (LE) is defined by Formula (1)^34^, where MW is the molecular weight in g/mol.


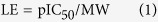


The LE for compound **5** is 0.010 for sEH and 0.0079 for FLAP. For **6** the results are 0.0065 (sEH) and 0.0059 (FLAP), indicating that as far as LE is concerned, **6** also constitutes a promising drug lead^35^.

In conclusion, the combination and application of two independently created pharmacophore model collections for the two pro-inflammatory targets allowed us to identify a completely novel and highly potent dual inhibitor of FLAP/sEH that will be further pharmacologically characterized. The synthesis of derivatives can be employed to experimentally verify the key structural elements for the activity on both enzymes. Moreover, a synthetic route for **5** was developed to provide more material for biological tests. A complete description of the synthesis is given in the Supplementary Information.

## Methods

### Molecular modeling

In ligand-based pharmacophore modeling, conformations of active molecules are computationally aligned and common pharmacophore features are placed where physicochemical functionalities overlap^36^. Conformations for this process were calculated with OMEGA^37^,^38^, which is implemented in LigandScout 3.12. The pharmacophore models were created using LigandScouts “create shared pharmacophore” function and optimized by adding an X-vol coat. Virtual screening was also performed in LigandScout, generating a 3D conformational library with 25 conformers per entry under omega-fast settings from the SPECS virtual library (202,920 compounds, version 2012). Default settings were used for virtual screening. Clustering the virtual hits for structural diversity was performed via ECFP4 in Accelrys’ Discovery Studio using the “find diverse molecules” protocol, using default settings, and requiring the program to cluster the ligands into ten groups.

### Biological testing

#### Cells

Human neutrophils were freshly isolated from leukocyte concentrates obtained from the Institute of Transfusion Medicine, University Hospital Jena, as described^39^. Donors (healthy adult volunteers) were informed about the aim of the study and gave written consent. The protocol for experiments was approved by the ethical commission of the University Hospital in Jena. All methods were performed in accordance with the relevant guidelines and regulations. Briefly, neutrophils were isolated by dextran sedimentation, centrifugation on lymphocyte separation medium (LSM 1077, PAA, Coelbe, Germany) and hypotonic lysis of erythrocytes. Neutrophils were resuspended in PBS containing glucose (0.1%) to a final cell density of 5 × 10^6^ cells/ml.

#### 5-LO purification and cell-free 5-LO activity test

*E. coli* (BL21) was transformed with pT3–5-LO plasmid (kindly provided by Dr. Olof Radmark, Karolinska Institute, Stockholm, Sweden), and recombinant 5-LO protein was expressed at 30 °C as described^40^. Cells were lysed in 50 mM triethanolamine/HCl pH 8.0, 5 mM EDTA, 1 mM phenylmethanesulphonyl fluoride, soybean trypsin inhibitor (60 μg/ml), and lysozyme (1 mg/ml), homogenized by sonication (3 × 15 s), and centrifuged at 40,000 × g for 20 min at 4 °C. 5-LO was purified from the 40,000 × g supernatant (S40) on an ATP-agarose column. Aliquots of semi-purified 5-LO were diluted with ice-cold PBS containing 1 mM EDTA, pre-incubated with the test compounds or vehicle (0.1% DMSO) on ice for 10 min. 5-LO product formation was initiated by addition of 20 μM AA and the reaction was stopped after 10 min at 37 °C. 5-LO metabolites were analysed by RP-HPLC as described. 5-LO products include the all-trans isomers of LTB4 as well as 5(*S*)-hydroperoxy-6-*trans*-8,11,14-*cis*-eicosatetraenoic acid (5-HPETE) and its corresponding alcohol 5(*S*)-hydroxy-6-*trans*-8,11,14-*cis*-eicosatetraenoic acid (5-HETE)^41^.

#### 5-LO product formation in intact neutrophils

Freshly isolated neutrophils (5 × 10^6^ cells/ml) were suspended in PGC buffer (PBS pH 7.4, CaCl_2_ 1 mM, glucose 0.1%), pre-incubated with the test compounds or vehicle (0.1% DMSO) for 10 min at 37 °C and stimulated with 2.5 μM Ca^2+^-ionophore A23187 for additional 10 min at 37 °C. The reaction was stopped by one volume (1 ml) of MetOH and 5-LO products (LTB4 and its trans-isomers as well as 5-HPETE and 5-HETE) were analyzed by HPLC as described above.

#### sEH purification and activity test

Human recombinant sEH was expressed and purified as described^21^,^42^. Briefly, Sf9 insect cells were cultured in suspension at 27 °C and infected with the recombinant baculovirus (kindly provided by Dr. B. Hammock, University of California, Davis, CA). After 72 h, cells were harvested and disrupted in buffer (50 mM NaHPO_4_, pH 8, 300 mM NaCl, 10% glycerol, 1 mM EDTA, 1 mM PMSF, 10 μg/ml leupeptin, and 60 μg/ml soybean trypsin inhibitor) by sonication (3 × 10 sec at 4 °C) and centrifuged for 1 h at 100,000 × g and 4 °C. sEH was purified from the supernatant by affinity chromatography utilizing benzylthio-sepharose and elution by 4-fluorochalcone oxide in PBS containing 1 mM DTT and 1 mM EDTA. The eluted enzyme solution was dialyzed, concentrated using Millipore Amicon-Ultra-15 centrifugal filter units and wash buffer, and the purity was verified by SDS-PAGE.

Enzyme activity of sEH was determined by a fluorescence-based assay using the non-fluorescent compound PHOME (Cayman Chemical, Ann Arbor, MI), which is converted by sEH to the fluorescent 6-methoxy-naphtaldehyde. Test compound or vehicle were pre-incubated with sEH in assay buffer (25 mM Tris HCl, pH 7, 0.1 mg/ml BSA) for 10 min at room temperature. PHOME (50 μM) was added and incubated for 60 min in the dark. The reaction was stopped by ZnSO_4_ and the fluorescence was monitored (λ_em_ 465 nm, λ_ex_ 330 nm). Potential fluorescence or quenching by the tested compounds was determined by adding the tests compounds to the assay in the absence of sEH enzyme, and any autofluorescence was subtracted from the read out when applicable; fluorescence quenching by the test compounds was not observed.

### Statistics

Data are expressed as mean ± S.E.M. IC_50_ values were calculated by nonlinear regression using GraphPad Prism Version 6 software (San Diego, CA) one site binding competition. Statistical evaluation of the data was performed by one-way ANOVA followed by a Bonferroni post hoc test for multiple comparison. A p value < 0.05 (*) was considered significant.

## Additional Information

**How to cite this article:** Temml, V. *et al*. Discovery of the first dual inhibitor of the 5-lipoxygenase-activating protein and soluble epoxide hydrolase using pharmacophore-based virtual screening. *Sci. Rep.* **7**, 42751; doi: 10.1038/srep42751 (2017).

**Publisher's note:** Springer Nature remains neutral with regard to jurisdictional claims in published maps and institutional affiliations.

## Supplementary Material

Supplementary Information

## Figures and Tables

**Table 1:** Overview on test substances and biological test results.

Compound	Model FLAP	Model sEH	5-LO product formation in intact neutrophils			sEH activity
			Remaining activity (% of control ± SEM) at			
			1 μM	10 μM	IC_50_ [μM] FLAP	IC_50_ [μM] sEH
1	Flap1	n.f.^*a*^	104.4 ± 2.8	47.8 ± 1.6	~10	n.d.^*b*^
2	Flap1	n.f.	102.0 ± 4.6	59.9 ± 2.0	>10	n.d.
3	Flap1	n.f.	95.1 ± 3.6	23.5 ± 1.1^*^	>1	n.d.
4	Flap2	n.f.	67.5 ± 3.1	6.6 ± 3.0	>1	n.d.
5	Flap2	1 and 8	1.6 ± 1.6^**^	1.7 ± 1.7^**^	0.2 ± 0.04	0.02 ± 0.007
6	Flap2	2, 3 and 4	83.5 ± 3.8	71.3 ± 11.8	18 ± 0.5	11.4 ± 0.5
7	Flap2	2	102.2 ± 10.1	105.3 ± 3.2	>10	>100
8	Flap2	2	85.4 ± 4.8	56.7 ± 4.2*^c^*	>10	>30
9	Flap2	4	88.5 ± 5.5	81.9 ± 8.1	>10	>100
10	Flap2	1 and 4	112.6 ± 8.9	109.6 ± 7.2	>10	3.0 ± 0.3
11	Flap2	3 and 4	97.3 ± 3.8	94.6 ± 2.5	>10	4.7 ± 0.2
12	Flap1	n.f.	106.0 ± 6.3	109.8 ± 5.5	>10	n.d.
13	Flap1	n.f.	115.3 ± 7.9	86.2 ± 10.2	>10	n.d.
14	Flap1	n.f.	107.5 ± 0.3	105.7 ± 1.3	>10	n.d.
15	Flap1	n.f.	95.8 ± 0.8	63.7 ± 6.5	>10	n.d.
16	Flap2	n.f.	93.2 ± 4.4	90.1 ± 13.0	>10	n.d.
17	Flap1	n.f.	107.6 ± 2.7	107.4 ± 3.0	>10	n.d.
18	Flap1	n.f.	105.4 ± 7.7	106.6 ± 4.2	>10	n.d.
19	Flap1	n.f.	110.3 ± 4.5	108.1 ± 5.6	>10	n.d.
20	Flap2	n.f.	84.2 ± 6.3	71.9 ± 4.5	>10	n.d.

*p < 0.05, **p < 0.01, one-way ANOVA followed by Bonferroni post hoc test.

^*a*^n.f.-not found; ^*b*^n.d.- not determined; *^c^*not tested for direct 5-LO inhibition.
