# Supplementary material for: Discovery of the first dual inhibitor of the 5-lipoxygenase-activating protein and soluble epoxide hydrolase using pharmacophore-based virtual screening
Source: Sci Rep. 2017 Feb 20;7:42751. doi: 10.1038/srep42751 (PMC5317001; doi:10.1038/srep42751)
Supplement: Supplementary Information [file srep42751-s1.pdf]

Discovery of the first dual inhibitor of the 5-lipoxygenase-activating protein and soluble epoxide hydrolase using pharmacophore-based virtual screening

Veronika Temml, Ulrike Garscha, Erik Romp, Gregor Schubert, Jana Gerstmeier, Zsofia Kutil, Barbara Matuszczak, Birgit Waltenberger, Hermann Stuppner, Oliver Werz, Daniela Schuster

Supporting Information

Table S1: Dataset of FLAP inhibitors from literature

Number	Chemical Structure	CAS	Activity	Reference
**S1**	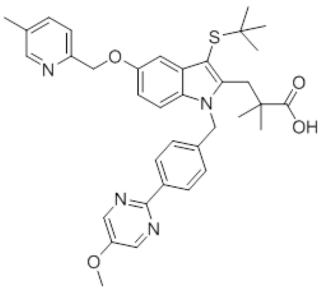	1233114-22-1	IC_{50} = 2 nM	Stock *et al*[1]
**S2**	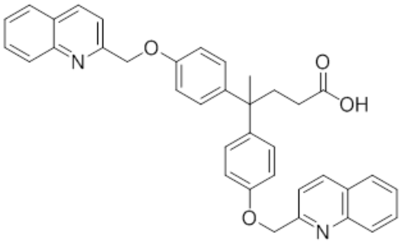	189498-56-4	IC_{50} = 20 nM	Kolasa *et al*[2]
**S3**	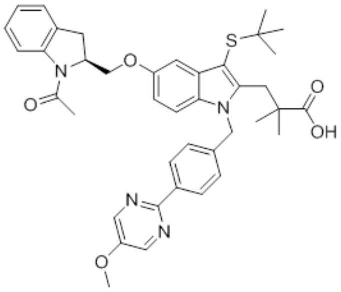	1206880-66-1	IC_{50} = 154 nM	Musiyenko *et al*[3]
**S4**	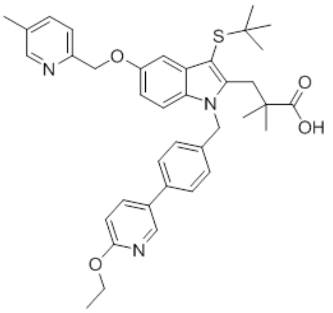	936350-00-4	IC_{50} = 2.9 nM	Stock *et al*[4]

**S5**	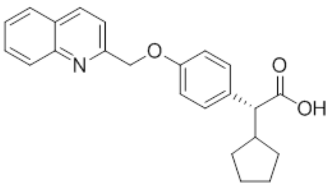	128253-31-6	IC_{50} = 58 nM	Stock *et al*[4]
**S6**	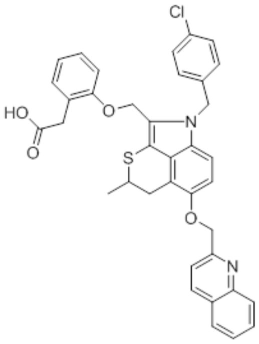	161447-90-1	IC_{50} = 32 nM	Stock *et al*[4]
**S7**	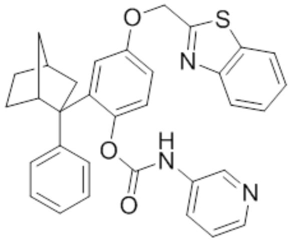	836647-96-2	IC_{50} = 2.8 nM	Macdonald *et al*[5]
**S8**	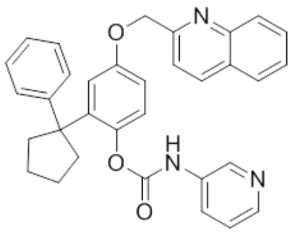	1025421-64-0	IC_{50} = 2.4 nM	Macdonald *et al*[5]
**S9**	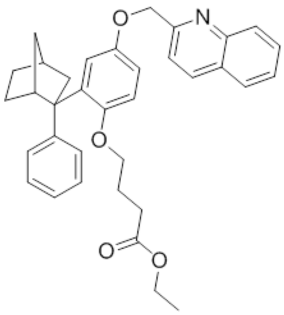	1025421-10-6	IC_{50} = 3.7 nM	Macdonald *et al*[5]

**S10**	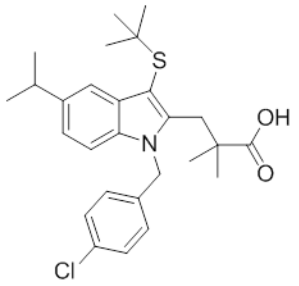	118414-82-7	IC_{50} = 1727 nM	Lorrain *et al*[6]
**S11**	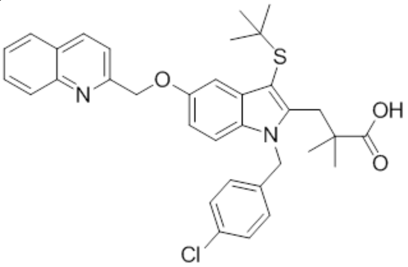	136668-42-3	IC_{50} = 1.6 nM	Brideau *et alDa*[7]

Pharmacophore model refinement:

FLAP1: The original automatically generated shared feature pharmacophore based on **10** and **11** consisted of four hydrophobic features, three hydrogen bond acceptor features (HBA), three aromatic features, and a negative ionizable feature. The pharmacophore was optimized by deleting two hydrophobic features, one aromatic, and two HBA features. The resulting model was constituted of two adjoining aromatic features and a negative ionizable feature in close vicinity to a HBA. To refine the model and to render it more specific, feature sizes were slightly altered and an exclusion volume (X-vol) coat was added.

FLAP2: In this case, the originally automatically generated shared feature pharmacophore model was left almost unmodified, only feature definitions were adapted and an exclusion volume coat was added for higher restrictivity. The model consisted of two hydrophobic features, two aromatic features, and two adjoining HBA features. These two HBA features are a common motif for sEH inhibitors, since they often contain a urea moiety.[8]

Table S2: Virtual hits selected for experimental testing.

Structure	code	Model FLAP	Models sEH
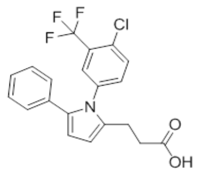	**1**	Flap1	not found
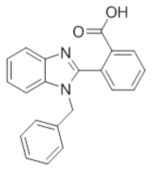	**12**	Flap1	not found
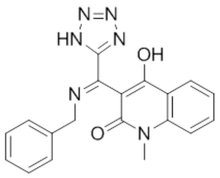	**18**	Flap1	not found
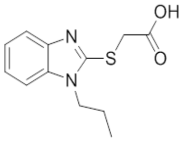	**19**	Flap1	not found
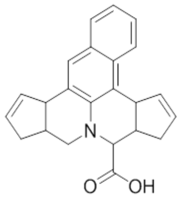	**2**	Flap1	not found
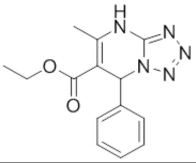	**13**	Flap1	not found
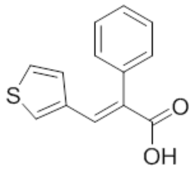	**14**	Flap1	not found

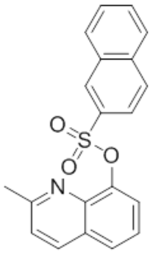	**15**	Flap1	not found
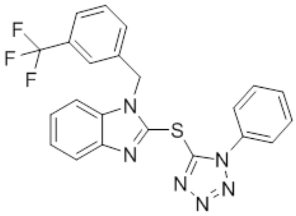	**3**	Flap1	not found
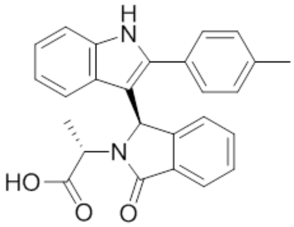	**17**	Flap1	not found
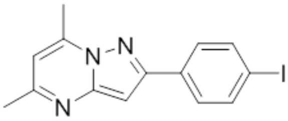	**4**	Flap2	not found
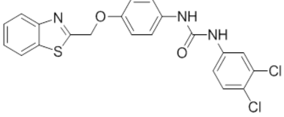	**5**	Flap2	Model 1 and 8

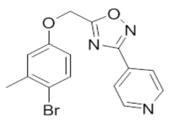	**7**	Flap2	Model 2
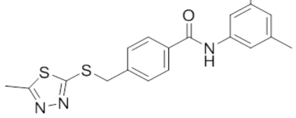	**10**	Flap2	Model 1 and 4
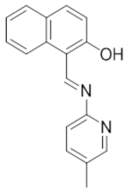	**20**	Flap2	not found
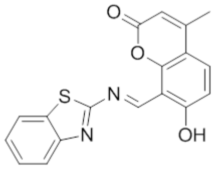	**8**	Flap2	Model 2
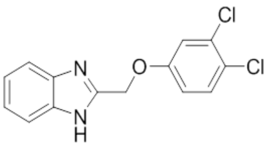	**11**	Flap2	Model 3 and 4
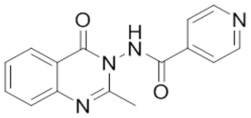	**9**	Flap2	Model 4
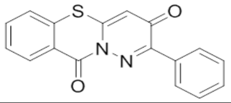	**16**	Flap2	not found

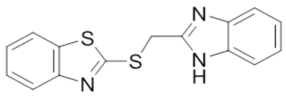	**6**	Flap2	Model 2,3 and 4

Description of the active hits and their orientation in the pharmacophore models

Pharmacophore model FLAP6 was able to identify two compounds that displayed substantial activity: **1** (50 % inhibiton at 20 μM) and **3**(75 % inhibition at 20 μM) (Figure S1).

According to the pharmacophore model, the functionalities responsible for the activity in compound **1** are the acid moiety that covers the HBA and negative ionizable feature and the two aromatic rings attached to the central pyrrole, which map into the two hydrophobic features. The two aromatic features are in this case mapping on only one aromatic ring, suggesting that these features could also be merged into one to further optimize the pharmacophore.

In compound **3** by contrast the two aromatic (and one hydrophobic) features are mapped by a benzimidazole, consisting of two condensed rings, a structural motif that was present in all molecules of the original dataset. The second hydrophobic feature is covered with a substituted phenyl ring, while the HBA and negative ionizable feature are in this case mapped on a tetrazole ring. The molecule also contains another phenyl group that is not mapped on a feature of the model but also doesn't clash with the X-vol coat. Considering that most FLAP inhibitors are comparatively large molecules and many smaller molecules in our hitlist turned out to be inactive, this ring likely also contributes to the compounds activity.

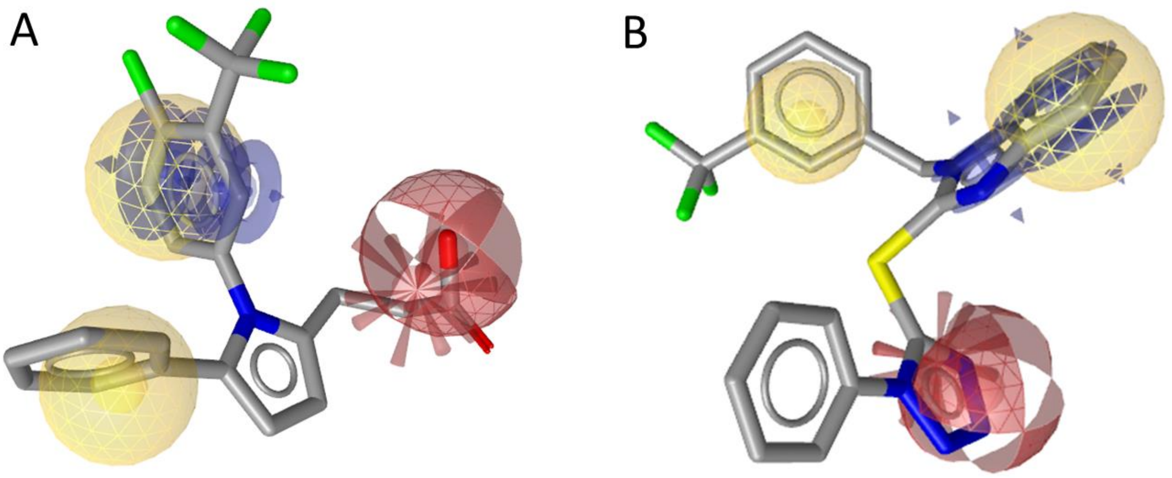

***Figure S1**: The active molecules **1** (A) and **3** (B) in the pharmacophore model FLAP1.*

4 consists of a pyrazolopyrimidine linked to an iodine substituted phenyl. **4** displayed an inhibition of 95 % at 10 µM and is much smaller than any known FLAP inhibitor. A methyl group and the phenyl moiety cover the hydrophobic features. The aromatic features are mapped on the phenyl group and pyrazolopyrimidine, while the two aromatic nitrogen atoms are mapped on the HBA features (Figure S2).

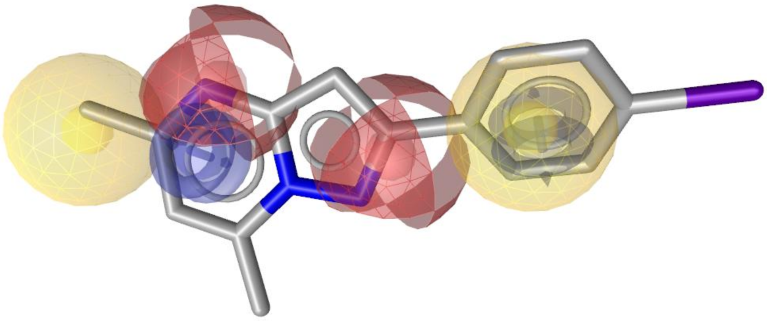

Figure S2: *FLAP inhibitor **4** n the model FLAP2*

4 was mapped into a pharmacophore for sEH (model 4, Figure S2). The two hydrophobic features of the model are mapped with a phenyl ring and a methyl group, while the HBD and HBA feature are covered by an amide group. The group is similar to the urea moiety, a typical sEH binding motif (Figure S3 (A)).

The same pharmacophore also retrieved **11**. In this case, the two hydrophobic features are mapped on a phenyl ring and on a benzimidazole. The HBA and HBD features are also mapped on the two nitrogen atoms of the benzimidazole, proposing a novel scaffold with the ability to inhibit sEH (Figure S3 (B)).

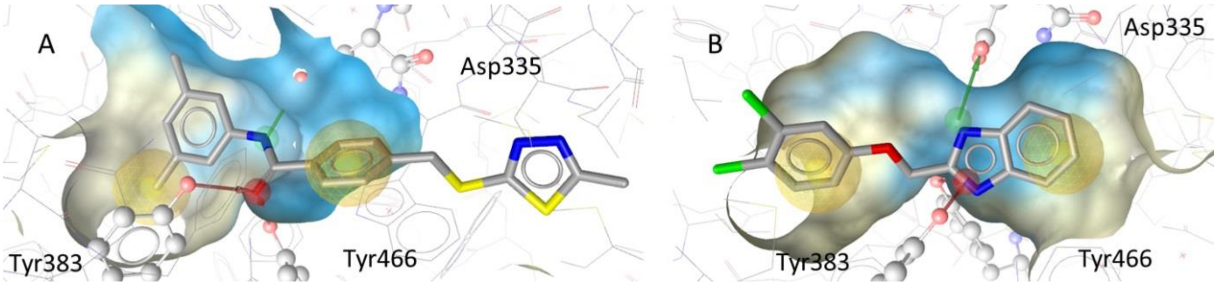

Figure S3: *Compound **10** (A) and **11**(B) in the binding pocket of the sEH crystal structure 3i1y.*[9]

Synthesis of 5

5 could be prepared in a three step procedure as shown in figure S4. In the first step, reaction of 2-chloromethylbenzothiazole with 3-methyl-4-nitrophenol in acetone in the presence of caesium carbonate, sodium carbonate and potassium iodide at reflux afforded the 3-methyl-4-

nitrophenoxymethyl substituted heteroarene. Reduction of the nitro function with hydrogen and Raney-Nickel as catalyst and subsequent reaction with 3,4-dichlorophenylisocyanate led to the target compound **5.**

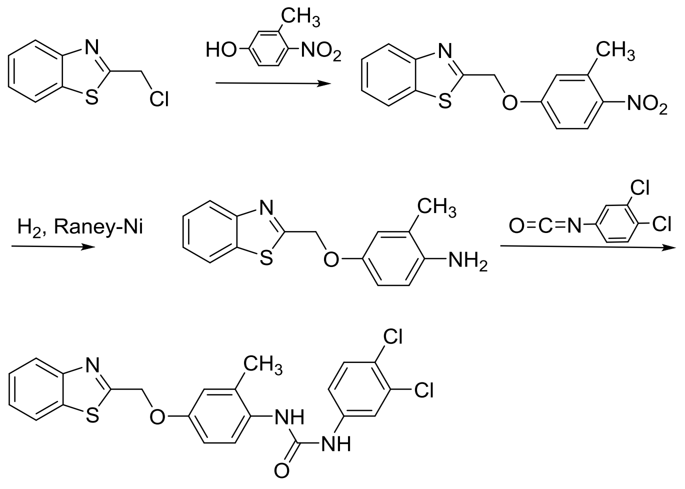

Figure S4: Synthesis of **5**

Experimental Part

Reactions were monitored by TLC using Polygram® SIL G/UV_{254} (Macherey-Nagel) plastic-backed plates (0.25 mm layer thickness), column chromatography was performed using silicagel 60 (40-63 µm). The yields are not optimised. Melting points were determined with a Kofler hot-stage microscope (Reichert) and are uncorrected. IR spectra were recorded on a Bruker ALPHA FT-IR apparatus equipped with a Platinum ATR module. ^{1}H-NMR spectra were recorded on a Varian Gemini 200 spectrometer (^{1}H: 199.98 MHz). The centre of the solvent multiplet (DMSO-d_6) was used as internal standard (chemical shifts in δ ppm), which was related to TMS with δ 2.49 ppm. Elemental analyses were performed by Mag. J. Theiner, 'Mikroanalytisches Laboratorium', Faculty of Chemistry, University of Vienna, Austria

2-(3-Methyl-4-nitrophenoxymethyl)benzothiazole (CAS Registry Number 197364-74-2)

The compound was synthesized according to a procedure described in the literature:[10] A mixture of 2-chloromethylbenzothiazole (3 mmol, 551 mg), of 3-methyl-4-nitrophenol (3 mmol, 459 mg), caesium carbonate (3 mmol, 977 mg), sodium carbonate (3 mmol, 318 mg), potassium iodide (1 mmol, 150 mg) and acetone (40 mL) was heated at reflux until the starting material was consumed as determined by tlc (ca. 6 h). The mixture was filtered and the resulting solution was partially concentrated *in vacuo*. The crystals thus formed were isolated, washed with acetone,

and dried to give 663 mg (74 % yield) of the pure product as light orange crystals, mp 146-148 °C.

IR*:* no characteristic *a*bsorption bands. ^{1}H-NMR (DMSO-d$_6$) δ [ppm]: 8.15-8.00 (m, 3H, arene H), 7.58-7.42 (m, 2H, arene H), 7.23 (d, J = 2.8 Hz, 1H, arene H), 7.14 (dd, J = 9.0, 2.8 Hz, 1H, arene H), 5.74 (s, 2H, CH_2), 2.54 (s, 3H, CH_3).

2-(4-Amino-3-methylphenoxymethyl)benzothiazole (CAS Registry Number 197364-75-3)

A mixture of the nitro compound (918 mg, 3.06 mmol), in ethanol (250 mL) was reduced on a Parr hydrogenator at 40 psi using Raney nickel catalyst until the reduction was complete (determined by tlc, ca. 10 hrs.). Then, the mixture was filtered to remove the catalyst and the filtrate was evaporated under reduced pressure. The product thus obtained was purified by column chromatography (eluent: dichloromethane/ethyl acetate = 1/1) to give 720 mg (87 %) of desired product as a beige solid, mp 109-111 °C.

IR: 3424, 3296, 3177. ^{1}H-NMR (DMSO-d$_6$) δ [ppm]: 8.09 (dd, J = 7.6, 1.0 Hz, 1 H, arene H), 7.99 (dd, J = 7.0, 1.0 Hz, 1 H, arene H), 7.55-7.39 (m, 2H, arene H), 6.75-6.65 (m, 2H, arene H), 6.53 (d, J = 8.2 Hz, 1H, arene H), 5.41 (s, 2H, CH_2), 4.48 (s br, 2H, NH_2), 2.02 (s, 3H, CH_3).

5: *N*-[4-(2-benzothiazolylmethoxy)-2-methylphenyl]-*N'*-(3,4-dichlorophenyl)-urea (CAS Registry Number 724453-98-9)

A solution of 3,4-dichlorophenylisocyanate (1.65 mmol, 310 mg) in dry tetrahydrofuran (2 mL) was added dropwise to a solution of the amino compound (1.50 mmol, 406 mg) in dry tetrahydrofuran (15 mL) and the resulting mixture was stirred at room temperature for 2 h. Then, diethyl ether was added and the solid obtained was collected, washed with tetrahydrofuran, and dried. The product was purified by treatment with acetone to yield 594 mg (86 %) of the product as a white solid, mp 253-253.5 °C.

IR [cm^{-1}] 3274, 1639. ^{1}H-NMR (DMSO-d$_6$) δ [ppm]: 9.84 (s, 1H, *D_2O exchangeable*, NH), 8.31 (s, 1H, *D_2O exchangeable*, NH), 8.13-8.09 ('m', 1H, arene H), 8.03-7.99 ('m', 1H, arene H), 7.87 (d, J = 2.2 Hz, 1H, arene H), 7.58-7.40 (m, 4H, arene H), 7.31 (dd, J = 8.9, 2.5 Hz, 1H, arene H), 6.97-6.86 (m, 2H, arene H), 5.55 (s, 2H, CH_2), 2.22 (s, 3H, CH_3). Calcd for $C_{22}H_{17}Cl_2N_3O_2S$: C, 57.65, H, 3.74; N, 9.17; S, 7.00. Found: C, 57.63, H, 3.72, N, 9.21; S, 6.93.

References

1 Stock, N. *et al.* 5-Lipoxygenase-activating protein inhibitors. Part 3: 3-{3-tert-Butylsulfanyl-1-4-(5-methoxy-pyrimidin-2-yl)-benzyl -5-(5-met hylpyridin-2-ylmethoxy)-1H-indol-2-yl -2,2-dimethyl-propionic acid (AM643)-A potent FLAP inhibitor suitable for topical administration. *Bioorganic & Medicinal Chemistry Letters* **20**, 4598-4601, doi:10.1016/j.bmcl.2010.06.011 (2010).

2 Kolasa, T. *et al.* Symmetrical bis(heteroarylmethoxyphenyl) alkylcarboxylic acids as inhibitors of leukotriene biosynthesis. *Journal of Medicinal Chemistry* **43**, 3322-3334, doi:10.1021/jm000180n (2000).

3 Musiyenko, A. *et al.* A Novel 5-Lipoxygenase-Activating Protein Inhibitor, AM679, Reduces Inflammation in the Respiratory Syncytial Virus-Infected Mouse Eye. *Clinical and Vaccine Immunology* **16**, 1654-1659, doi:10.1128/cvi.00220-09 (2009).

4 Stock, N. S. *et al.* 5-Lipoxygenase-Activating Protein (FLAP) Inhibitors. Part 4: Development of 3- 3-tert-Butylsulfanyl-1- 4-(6-ethoxypyridin-3-yl)benzyl -5-(5-methylpy ridin-2-ylmethoxy)-1H-indol-2-yl -2,2-dimethylpropionic Acid (AM803), a Potent, Oral, Once Daily FLAP Inhibitor. *Journal of Medicinal Chemistry* **54**, 8013-8029, doi:10.1021/jm2008369 (2011).

5 Macdonald, D. *et al.* Substituted 2,2-bisaryl-bicycloheptanes as novel and potent inhibitors of 5-lipoxygenase activating protein. *Bioorganic & Medicinal Chemistry Letters* **18**, 2023-2027, doi:10.1016/j.bmcl.2008.01.105 (2008).

6 Lorrain, D. S. *et al.* Pharmacological Characterization of 3- 3-tert-Butylsulfanyl-1- 4-(6-methoxy-pyridin-3-yl)-benzyl -5-(pyridin -2-ylmethoxy)-1H-indol-2-yl -2,2-dimethyl-propionicAcid (AM103), a Novel Selective 5-Lipoxygenase-Activating Protein Inhibitor That Reduces Acute and Chronic Inflammation. *Journal of Pharmacology and Experimental Therapeutics* **331**, 1042-1050, doi:10.1124/jpet.109.158089 (2009).

7 Brideau, C. *et al.* Pharmacology of MK-0591 (3- 1-(4-Chlorobenzyl)-3-(t-butylthio)-5-(quinolin-2-yl-methoxy)-indol-2 -yl -2,2-dimethyl propanoic acid), a potent, orally active leukotriene biosynthesis inhibitor. *Canadian Journal of Physiology and Pharmacology* **70**, 799-807 (1992).

8 Waltenberger, B. *et al.* Discovery of Potent Soluble Epoxide Hydrolase (sEH) Inhibitors by Pharmacophore-Based Virtual Screening. *Journal of Chemical Information and Modeling* **56**, 747-762, doi:10.1021/acs.jcim.5b00592 (2016).

9 Eldrup, A. B. *et al.* Structure-Based Optimization of Arylamides as Inhibitors of Soluble Epoxide Hydrolase. *Journal of Medicinal Chemistry* **52**, 5880-5895, doi:10.1021/jm9005302 (2009).

10 Musser, J. H. *et al.* Leukotriene D4 antagonists and 5-lipoxygenase inhibitors. Synthesis of benzoheterocyclic [(methoxyphenyl)amino]oxoalkanoic acid esters. *Journal of Medicinal Chemistry* **30**, 400-405, doi:10.1021/jm00385a024 (1987).
